# What are the differences in protective characteristics of orthodontic mouthguards? An *in vitro* study

**DOI:** 10.1093/ejo/cjab023

**Published:** 2021-06-01

**Authors:** Claire Harrington, Gursharan Minhas, Spyridon N Papageorgiou, Martyn T Cobourne

**Affiliations:** 1 Department of Orthodontics, The Royal Surrey County Hospital, Guildford, Surrey, UK; 2 Centre for Craniofacial Development & Regeneration, Department of Orthodontics, Faculty of Dentistry, Oral & Craniofacial Sciences, King's College London, London, UK; 3 Clinic of Orthodontics and Pediatric Dentistry, Center of Dental Medicine, University of Zurich, Zurich, Switzerland

## Abstract

**Background:**

Orthodontic patients wearing fixed appliances are susceptible to traumatic dental injuries during contact-sport. This laboratory study investigated the protective qualities of orthodontic mouthguards using impact-testing to a typodont fitted with a fixed appliance through peak load transfer and retention of the mouthguard.

**Methods:**

Seven orthodontic mouthguards [three custom-made (Medium-CM, Heavy-CM, Heavy-pro-CM); three commercially-available mouth-formed (Shock-Doctor® Ultra Braces, Opro® Ortho-Gold Braces, Opro® Ortho-Bronze Braces) and a Shock-Doctor® Instant-Fit] were fitted to a maxillary arch typodont bonded with a fixed appliance and impact-tested using 0.5 or 1 Joule (J) energy via hockey-ball, cricket-ball or steel-ball projectile. A load-cell recorded peak load transfer through mouthguard to typodont with retention scored in a binary manner dependent upon any displacement following impact. Differences across mouthguards were calculated with ANOVA or Kruskal–Wallis test for normal and non-normal data, respectively. Post hoc comparisons across mouthguards were conducted via Dunnett's test with Sidak correction.

**Results:**

Only the three custom-made and Opro® Ortho-Gold Braces were not displaced by impact-testing. For these, Opro® Ortho-Gold Braces transferred the smallest load for 3/6 impact-tests, followed by Medium-CM. Heavy-pro-CM performed poorly, ranking penultimate or worst for all impact-tests. Significant differences were found between mouthguards for cricket-ball and steel-ball set-ups. The Opro® Ortho-Gold Braces performed better than the Heavy and Heavy-pro-CM for 0.5 J cricket-ball impact-test (*P* < 0.05), whilst Medium-CM performed better than Heavy-pro-CM. For 1 J cricket-ball, there were significant differences between Medium-CM and Heavy-pro-CM (*P* < 0.05). For 0.5 J steel-ball, the Medium-CM performed significantly better than both Heavy-pro-CM and Opro® Ortho-Gold Braces (*P* < 0.05), whilst Heavy-CM performed better than the Heavy-pro-CM (*P* < 0.05). For the 1 J steel-ball, Medium and Heavy-CM performed better than Opro® Ortho-Gold Braces (*P* < 0.05).

**Conclusions:**

Opro® Ortho-Gold and Medium-CM mouthguards offer the best protection for low-impact sports, whilst Medium or Heavy-CM mouthguards are recommended for high-impact sport.

## Introduction

Sport-related injuries are a global phenomenon with an estimated 40% of children and adolescents affected annually (1–3). Moreover, a significant number of sports injuries affect the dentition (4) with boys most commonly affected and a peak incidence occurring between 8 and 11 years of age (5). Sport-related dental injuries are often irreversible and can lead to long-term functional, aesthetic, and psychological problems, which can have a negative impact on oral health-related quality of life (6). It has been suggested that up to half of these injuries are preventable (7, 8) and wearing a mouthguard is an effective preventative strategy (9, 10).

Children and young adults undergoing orthodontic treatment with fixed appliances are also susceptible to sport-related injuries affecting the oro-facial region. However, the presence of a fixed orthodontic appliance can be a deterrent, with only 35% of orthodontic patients reported to routinely wear a mouthguard during sport (11). Fixed appliances complicate any potential injury through loss of brackets and attachments, archwire distortions, and unwanted tooth movement, making them a risk factor for sport-related oro-facial injury (4). In addition, the presence of an underlying malocclusion, particularly an increased overjet is also a risk factor for traumatic injury. The majority of UK hospital-based consultant orthodontists recommend custom-made mouthguards (12), which are thought to offer the best general protection and comfort for patients wearing fixed braces (13–16). However, there is little data from orthodontic patients or laboratory-based typodont studies in relation to their effectiveness. Traditionally these mouthguards are constructed by either vacuum or pressure-forming a thermo-plastic material, most commonly ethyl-vinyl-acetate (EVA) in layers over a cast dental impression. However, the close-fitting nature can potentially damage a fixed brace and compromise the longevity of the mouthguard as tooth movement is taking place. They also require a high-quality impression and construction of the mouthguard by an appropriately trained laboratory technician. For these reasons, commercial ‘over-the-counter’ mouth-formed orthodontic (and non-orthodontic) mouthguards are also available. They are inexpensive, convenient to buy, and usually only require immersion in hot water before adaptation to the teeth and soft tissues; however, problems can arise with adequate moulding of the mouthguard around fixed braces and the dentition. The design and construction of these mouthguards has evolved over the years and it has been suggested that they might have similar protective qualities to custom-made (17) although findings vary (13–15). A more recent commercial mouthguard has also become available for use with fixed braces. The Shock-Doctor® Braces Mouthguard (Fountain Valley, California, USA) is constructed from medical-grade silicone and adapts to the teeth and brace without the need for immersion in hot water. These mouthguards attempt to address the difficulties in adapting and moulding mouth-formed mouthguards and the problems that may arise from them having an inadequate fit.

The aims of this laboratory-based study were to compare protective qualities of commercial and custom-made orthodontic mouthguards using impact-testing with different projectiles to a typodont model fitted with a fixed orthodontic appliance through peak load transfer measurement and retention of the mouthguard after impact-testing. The null hypothesis was that there are no differences in the protection afforded by different orthodontic mouthguards when used in conjunction with fixed braces.

## Materials and methods

This laboratory investigation was carried out under local approval from King's College London.

### Primary and secondary outcomes

The primary outcome was peak load transfer through the test mouthguard after impact-testing with a specific projectile. The smaller the measured peak load the more effective the mouthguard at absorbing energy. Secondary outcomes included retention of the mouthguard during impact-testing with any displacement (partial or complete) recorded after the initial impact of the projectile.

### Typodont model

Nissan operative typodont jaw models (CON2001-UL-UP-FEM-32, Kyoto, Japan) were used to replicate the upper jaw. Fixed orthodontic appliances with a 0.022 × 0.028-inch slot (3M Unitek, USA) were bonded (Loctite Superglue, Henckel Corporation, USA) directly onto acrylic teeth anteriorly and via orthodontic bands to the first permanent molars. A 0.017 × 0.025-inch stainless steel archwire was ligated into the brackets with conventional elastomeric ligatures. Only the upper arch was used for testing. Typodonts were fitted with the mouthguards and clamped onto the load cell for impact-testing.

### Impact-testing

**Figure 1.**
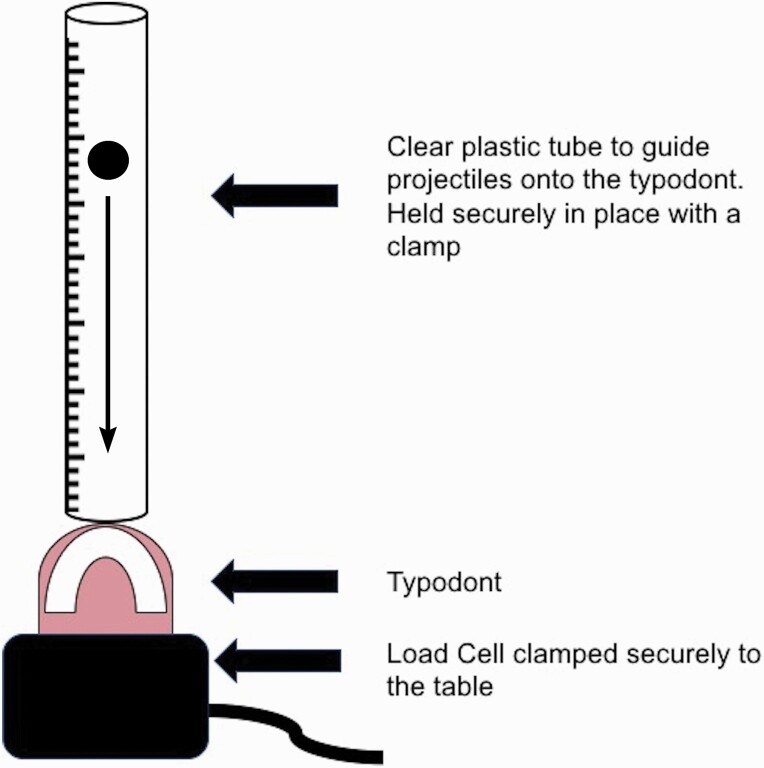
Experimental set-up for the impact testing.

A standardized impact-testing method was used in the experimental set-up (Figure 1) (14, 15) adapted to incorporate three carefully selected free-falling projectiles: 1. cricket-ball, 2. hockey-ball, and 3. steel-ball (Figure 1). These objects were initially impacted into typodonts without the mouthguard in place and then with each individually fitted mouthguard under test. The impacts were delivered through free-falling from heights calculated to provide 0.5 Joules (J) or 1.0 J of impact energy. A load cell (EnduraTEC, Model: 1010CCH-2.5K) was used to record peak load transfer for each mouthguard compared with no mouthguard (Newtons, N). Each typodont was impacted six times for each projectile at both 0.5 and 1.0 J. This was then replicated with each mouthguard in place and the average peak load transfer for each experimental set-up calculated. After each impact, the typodont was inspected for fractures or breakages.

For each experimental set-up, 42 measurements were taken. A total of seven mouthguards were tested with each undergoing six impacts per experiment. Peak load transfer was recorded for each one of these impacts and then an average taken.

### Retention of the mouthguard after impact-testing

Mouthguard retention and/or damage was assessed after the initial impact and rebound of the projectile with any displacement or movement of the mouthguard from the dentition scored in a binary manner. This visual analysis was carried out by a single assessor (CH).

The mouthguards that displaced during the testing meant inaccurate peak load transfer readings, due to potential energy loss during displacement. Therefore, the results from the three mouthguards that displaced following impact were not included for comparison. Mouthguards should be well-fitting and retentive in-order for them to be protective.

### Mouthguards

A total of seven orthodontic mouthguards were selected for testing. Three custom-made mouthguards (medium, heavy, and heavy-pro) and a selection of commercially-available mouthguards, including three mouth-formed: Shock-Doctor® Ultra Braces (Fountain Valley, CA, USA), Opro® Ortho Gold Braces and Opro® Ortho Bronze Braces (Hemel Hempstead, Hertfordshire, UK) and an instant-fit type Shock-Doctor® Braces. The custom-made mouthguards were constructed in a hospital maxillofacial orthodontic laboratory (Basingstoke and North Hampshire Hospital, Hampshire, UK) by a trained technician through pressure-forming ethylene-vinyl acetate (EVA) sheets over a cast dental arch of the typodont and fixed appliance (Drufomat SQ, Dreve-Dentamid GmbH, Unna, Germany). A laminating technique was used with the number of layers dependent on the type of mouthguard (medium custom-made mouthguard: 2 mm EVA followed by 4 mm EVA pressure-formed over the dental cast; heavy custom-made mouthguard: 2 mm EVA followed by hardened EVA force lines (Erkoflex 95) and another 4 mm EVA pressure-formed dental cast; heavy-pro custom-made mouthguard; 2 mm EVA followed by 0.8 mm hardened EVA insert (Erkodur S-Shell) followed by 4 mm EVA pressure-formed over the dental cast). The commercially available mouthguards are all designed and advertised for use with fixed orthodontic braces and are available for direct-purchase by patients. They were fitted to the typodont occlusion according to manufacturer instructions.

### Statistical analysis

Normality of the data was checked via visual inspection of histograms and formally with the Shapiro–Wilk test. Descriptive statistics included means and standard deviations (SD) for normally-distributed data and medians and interquartile ranges (IQR) for non-normally distributed data. Differences across mouthguards were calculated separately for each experimental set-up with a one-way analysis of variance (ANOVA) or Kruskal–Wallis test for normal and non-normal data, respectively. In cases of a statistically significant test, *post hoc* comparisons across mouthguards were conducted via Dunnett's test with a Sidak correction to significance level due to multiple testing. All analyses were performed in Stata 14.0 (StataCorp, College Station, TX) with a two-tailed alpha of 0.05. Primary data is deposited in the Zenodo data repository (18).

## Results

After each impact, the typodont and mouthguard were checked for the presence of any fractures or damage. Typodonts that were impact-tested with no mouthguard in-situ incurred multiple fractures to the teeth and fixed appliances, which resulted in distorted load transfer readings. These readings could not therefore be reliably used for comparison of impact-testing with a mouthguard in-situ. Use of a control was therefore abandoned and only direct comparisons between mouthguards were made. All the testing with mouthguards in-situ was associated with no damage to the typodonts or any of the mouthguards.

### Fit and retention of the mouthguard

The three custom-made mouthguards (medium, heavy, and heavy-pro) and Opro® Gold Braces mouth-formed mouthguard were all well-fitting, retentive to the typodont, and not displaced during impact-testing. The Opro® Bronze Braces and Shock-Doctor® Ultra Braces mouth-formed mouthguards and the Shock-Doctor® Braces instant-fit mouthguard were all poorly retentive. After every impact, the mouthguards were displaced from the typodont.

As a result of this poor retention, the Opro® Bronze Braces, Shock-Doctor® Ultra Braces and Shock-Doctor® Braces instant-fit mouthguards could not be used for comparison with those that were retentive due to the potential energy loss that would have occurred during displacement.

### Impact-testing

The descriptive statistics for the four retentive mouthguards that were subject to impact-testing are shown in Table 1. We further tested for statistical differences across all the mouthguards for each impact test. Significant differences were found for the cricket and steel-ball set-ups but none for the hockey-ball (Table 2). Where significant differences were found, a *post hoc* pairwise comparison was carried out for each impact test using Dunnett's test (Table 3). For the cricket-ball at 0.5 J the Opro® Gold Braces was found to perform better than the heavy and heavy-pro custom-made mouthguards (*P* = 0.03 and <0.001, respectively). The medium custom-made mouthguard performed better than the heavy-pro custom-made mouthguard (*P* < 0.04). The 1.0 J cricket-ball impact-testing showed a significant difference between the medium and the heavy-pro custom-made mouthguards (*P* < 0.002). For the steel-ball set-up at 0.5 J impact-testing, the medium custom-made mouthguard performed significantly better than both the heavy-pro custom-made and Opro® Gold Braces mouthguard (*P* < 0.001 and *P* = 0.04, respectively). The heavy custom-made mouthguard performed better than the heavy-pro custom-made mouthguard (*P* = 0.002). For the 1.0 J impact, the medium and heavy custom-made mouthguards performed better than the Opro® Gold Braces mouthguard (*P* = 0.003 and <0.001, respectively). All other comparisons between mouthguards showed no significant differences.

**Table 1.** Descriptive statistics for peak load transfer (*n*) for each mouthguard under test

Projectile; Energy (J)		Medium custom-made mouthguard	Heavy custom-made mouthguard	Heavy-pro custom-made mouthguard	Opro® Gold mouth-formed commercial mouthguard
Cricket ball; 0.5 J	Mean (SD)	288.1 (6.1)	294.9 (5.5)	305.3 (7.9)	272.5 (10.2)
Cricket ball; 1.0 J	Mean (SD)	470.4 (13.0)	482.0 (13.3)	504.1 (9.3)	477.8 (18.2)
Hockey ball; 0.5 J	Median (IQR)	323.9 (314.4–328.4)	348.2 (327.6–371.5)	324.8 (311.6–331.1)	319.1 (308.6–326.4)
Hockey ball; 1.0 J	Mean (SD)	546.1 (14.7)	535.7 (24.1)	547.4 (16.3)	526.1 (21.1)
Steel ball; 0.5 J	Median (IQR)	307.3 (305.1–309.0)	313.9 (304.1–318.8)	378.6 (372.6–380.4)	359.5 (345.2–364.2)
Steel ball; 1.0 J	Mean (SD)	575.1 (19.8)	563.6 (20.3)	603.3 (10.8)	653.5 (7.3)

Each experiment included a total of 42 measurements (six measurements per mouthguard type).

IQR, interquartile range; J, Joules; SD, standard deviation.

**Table 2.** *P* values for differences across mouthguards for each experiment

Ball; eergy	Test	*P* value
Cricket ball; 0.5 J	ANOVA	<0.001*
Cricket ball; 1.0 J	ANOVA	0.007*
Hockey ball; 0.5 J	Kruskal–Wallis	0.121
Hockey ball; 1.0 J	ANOVA	0.145
Steel ball; 0.5 J	Kruskal–Wallis	<0.001*
Steel ball; 1.0 J	ANOVA	<0.001*

*Significant *P* < 0.05.

**Table 3.** Post hoc pairwise mouthguard comparisons for each experiment

	Medium custom-made mouthguard	Heavy custom-made mouthguard	Heavy-pro custom-made mouthguard	Opro® Gold mouth-formed commercial mouthguard
Cricket ball; 0.5 J				
Medium custom-made mouthguard				
Heavy custom-made mouthguard	0.56			
Heavy-pro custom-made mouthguard	0.04*	0.45		
Opro Gold® mouth-formed commercial mouthguard	0.36	0.03*	<0.001*	
Cricket ball; 1.0 J				
Medium custom-made mouthguard				
Heavy custom-made mouthguard	0.56			
Heavy-pro custom-made mouthguard	0.002*	0.07		
Opro Gold® mouth-formed commercial mouthguard	0.63	0.97	0.05	
Steel ball; 0.5 J				
Medium custom-made mouthguard				
Heavy custom-made mouthguard	0.94			
Heavy-pro custom-made mouthguard	<0.001*	0.002*		
Opro Gold® mouth-formed commercial mouthguard	0.04*	0.09	0.5	
Steel ball; 1.0 J				
Medium custom-made mouthguard				
Heavy custom-made mouthguard	0.81			
Heavy-pro custom-made mouthguard	0.25	0.05		
Opro Gold® mouth-formed commercial mouthguard	0.003*	<0.001*	0.29	

Results are presented as *P* values with Sidak correction.

*Significant values (*P* < 0.05) are in bold.

**Figure 2.**
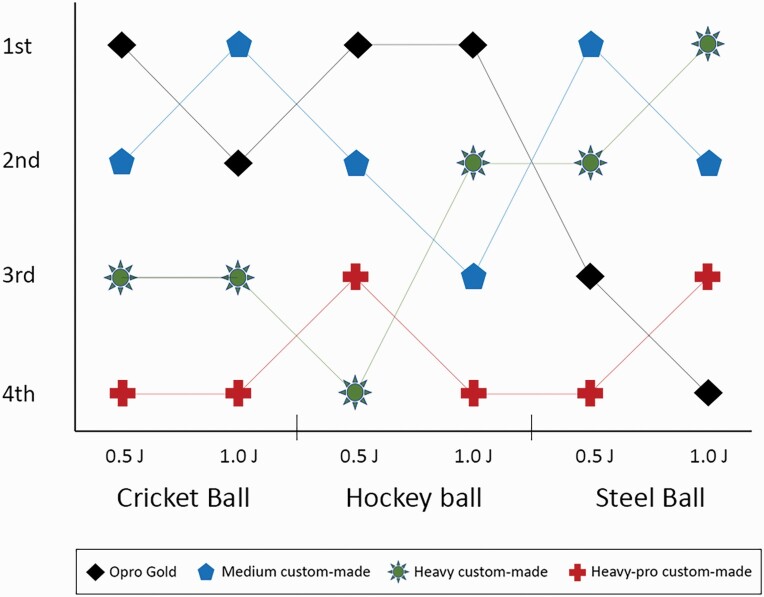
Ranking of all mouthguards for each projectile and energy level.

Finally, the mouthguards were ranked on the basis of load transfer during the impact-testing (Figure 2). Briefly, the Opro® Gold Braces mouthguard performed best of all, transferring the smallest load for three out of the six impact experiments (hockey-ball 0.5 J and 1.0 J; cricket-ball 0.5 J). This was followed by the medium custom-made mouthguard (performing best for steel-ball 0.5 J and cricket-ball 1.0 J). The heavy custom-made mouthguard performed best only for the 1.0 J steel-ball experiment. The heavy-pro custom-made mouthguard performed worst out of all the mouthguards, ranking the worst in four out of six impact-tests and in penultimate position for the hockey-ball 0.5 J and steel-ball 1.0 J.

## Discussion

This investigation compared the protective qualities of commercial and custom-made orthodontic mouthguards using a typodont model fitted with fixed appliances through peak load transfer after impact-testing with different projectiles. Impact objects were selected on the basis of similarity to those used in previous testing scenarios and representing popular sports played globally, whilst each model was impacted six times for comparability with other studies. The number of impacts and energy used in previous investigations ranges from three to ten times with 0.5 or 1 J loads, respectively (17, 19, 20). A steel-ball was also used to assess mouthguard performance following impact with increasing hardness, reflecting impacts such as a hockey stick or other sports equipment.

This increasing hardness was important for evaluating the protectiveness of the heavy and heavy-pro custom-made mouthguards, which are designed and advertised specifically for higher-impact sports. These mouthguards include a hardened insert or force lines within the laminated sheets although data on the energy absorption effectiveness of this design are conflicting (21, 22). Medium custom-made mouthguards are recommended for contact sports that do not encounter hard equipment (such as rugby football). Heavy custom-made mouthguards are recommended for sports where hard equipment, such as a bat or ball but direct full-on contact is less likely to be encountered (lacrosse, squash, and cricket). Heavy-pro custom-made mouthguards are recommended for professional sports players or players moving at speed who may encounter a hard weapon, ball or full contact (ice hockey and street hockey).

Mouthguards that displaced during impact-testing were the Opro® Bronze Braces, Shock-Doctor® Ultra Braces, and Shock-Doctor® Braces. The presence of displacement was highly relevant for peak load transfer measurements because for effective energy absorption the mouthguard needs to be retained on the dentition. The Opro® Bronze Braces mouthguard does not include the same design features as the Opro® Gold Braces mouthguard, which may have resulted in the poor retention. These unique design features include a brace bumper and shortened fins that are slim rib-like structures (a modification of Opro's® patented fin technology), which ultimately resulted in the Opro® Gold Braces mouthguard having a fit that was subjectively judged to be superior. The Shock-Doctor® Braces mouthguard has a special ‘ortho-channel’ and in combination with its construction from medical grade silicone, is claimed to be sufficiently flexible to adapt around the brackets and teeth. This was not evident from our experiments; the mouthguard although flexible, was found to lack retention over the fixed appliance and teeth during impact-testing. However, a combination of no saliva being present in the test set-up and intra-oral temperature not being replicated could have affected the resulting fit. The Shock-Doctor® Ultra Braces mouthguard was bulky in comparison to the other self-adapting mouthguards, due to the triple-layer construction. The flexible outer shell, internal core, and gel-fit lining as described by the manufacturers, did not seat fully around the fixed appliances and there was insufficient sulcus extension. This may have contributed to the lack of retention compared to the other commercially-available mouthguards, also combined with lack of saliva and replication of intra-oral temperature. The mouthguards that displaced during the testing meant inaccurate peak load transfer readings, due to potential energy loss during displacement. Therefore, the results from the three mouthguards that displaced the following impact were not included for comparison. Mouthguards should be well-fitting and retentive in-order for them to be protective.

The mouthguards that did not displace were the Opro® Gold Braces and the custom-made mouthguards (medium, heavy, and heavy-pro). The medium mouthguard generally performed best out of all the custom-made mouthguards. It performed better than the heavy-pro in every experimental set-up and this was significant for three (steel-ball 0.5 J, cricket-ball both 0.5 J and 1.0 J). There were no differences between the heavy and medium custom-made mouthguards, suggesting that they both afford similar protective qualities regardless of being reinforced with hardened force lines. The heavy and heavy-pro custom-made mouthguards are specially designed for higher-impact sports. Interestingly, the heavy-pro custom-made mouthguard performed worse than the medium and heavy. However, there was no significant difference for the highest impact simulation with the steel ball at 1 J impact energy. The medium and the heavy custom-made mouthguards did show a significant difference in their performance compared to the heavy-pro at 0.5 J steel ball impact. These are important findings because the steel ball replicates a higher impact situation suggesting that the medium custom-made mouthguard is as protective as mouthguards that are reinforced with hardened force lines and EVA shell inserts. These results do not support the idea that a hard insert provides more protection and conversely suggest that in some scenarios, less energy is absorbed (21, 22). The literature suggests that custom-made mouthguards confer greater protective qualities than the commercially-available mouthguards, although some more recent testing has shown that they are equally protective (17). In this investigation, the Opro® Gold Braces mouthguard performed best in three of six experimental set-ups. However, when compared to the medium custom-made mouthguard, they were found to be equally effective for both the cricket and hockey ball impact-testing. In the testing that represented higher-impact sports situations, the Opro® Gold Braces mouthguard did not perform as well, with the medium custom-made mouthguard performing significantly better in both steel ball experiments and the heavy custom-made mouthguard also significantly better in the 1.0 J steel ball experiment. Therefore, conclusions can be drawn that the Opro® Gold Braces mouthguard afforded similar protective qualities to custom-made mouthguards in sports where a hardened equipment is not likely to be encountered. But there is still a huge reliance on the wearer adequately fitting this mouthguard themselves. An advantage of the custom-made mouthguard is that it is not dependent on how good the wearer and/or parent is at fitting it and is constructed by trained professionals from an impression representing the exact intra-oral anatomy.

There were several limitations to this study. The impact energies used in this investigation are only a small example of potential impact energies in a sporting environment. If we were to use these types of high-impact energies, the typodonts would have fractured and been destroyed, which was not the aim of this investigation. Moreover, the use of pressure sensors associated with individual teeth might be considered as part of an improved experimental design for future studies. The fixed appliance set-up consisted of a commonly used pre-adjusted edgewise appliance system but testing was limited to a only a 0.017 × 0.025-inch dimension rectangular archwire to minimize the number of variables. However, different composition and dimension archwires may have influenced different performances. Moreover, the typodont dentition was not composed of real teeth and the brackets were glued rather than fixed using conventional etching and bonding. All these factors may have influenced performance. In addition, this was a laboratory study conducted using a typodont. In the real world, other peri-oral tissues may protect or worsen the injuries received. It is unknown whether displacement of the gum shield on impact is important. The tongue and mandibular teeth are likely to reposition the gum shield after injury. If however, displacement of the gum shield during an injury leads to further force or injuries to the dentition then this would be a key finding. Whilst the lips, if covering the teeth at the time of injury may reduce the force transmitted to the teeth, there is likely to be significant soft tissue injury if they become embedded into the fixed appliance. All these factors will influence performance.

The intra-oral environment was not replicated in this *in vitro* investigation and therefore the implications of saliva, intra-oral temperature, and suction may have aided in the stability of the mouthguard. We were also unable to test the new type of ‘instant fit’ technology mouthguard (Shock Doctor® Braces), which requires no modification in order to fit it because the mouthguard routinely displaced during impact-testing. Furthermore, fit and retention of the mouthguards was tested *in vitro* using only a maxillary model. In normal use, mouthguards might be better stabilized intraorally through the occlusion, with athletes stabilising the mouthguard during sport through biting between the jaws (23). Moreover, although this investigation did analyse fit and retention it did not address the comfort and wearability of the mouthguard from the sportspersons point-of-view. It is important to remember that even though the mouthguard may be protective, if it is not comfortable, the wearer will have difficulty using it.

## Conclusions

For the hockey ball impact-testing no significant differences between mouthguards were established. However, for cricket ball and steel ball impact-testing significant differences were found between custom-made mouthguards and the Opro® Gold Braces mouthguard.

1. Opro® Gold Braces and medium custom-made mouthguards afford similar protective qualities in lower-impact situations and are recommended for sports where hard equipment is unlikely to be encountered (non-stick sports);
2. Medium custom-made mouthguards performed well across all impact-testing and especially for high-impact situations, and
3. Custom-made mouthguards without any hardened inserts (medium custom-made mouthguard) performed better than those with hardened EVA shell inserts (heavy-pro custom-made mouthguard) across all impact-testing.

## Data Availability

The data underlying this article are available at Zenodo: http://doiorg/105281/zenodo4058479 [19].
